# A Combination of 5-(3′,4′-Dihydroxyphenyl)-γ-Valerolactone and Curcumin Synergistically Reduces Neuroinflammation in Cortical Microglia by Targeting the NLRP3 Inflammasome and the NOX2/Nrf2 Signaling Pathway

**DOI:** 10.3390/nu17081316

**Published:** 2025-04-10

**Authors:** Emma Marcolin, Chiara Chemello, Anna Piovan, Massimo Barbierato, Paolo Morazzoni, Eugenio Ragazzi, Morena Zusso

**Affiliations:** 1 Department of Pharmaceutical and Pharmacological Sciences, University of Padua, 35131 Padua, Italy; emma.marcolin.1@phd.unipd.it (E.M.); chiara.chemello@studenti.unipd.it (C.C.); anna.piovan@unipd.it (A.P.); silverflute.silverflute@gmail.com (M.B.); eugenio.ragazzi@unipd.it (E.R.); 2 Nutraceutical Division, Distillerie Umberto Bonollo S.p.A., 35035 Mestrino, Italy; paolo.morazzoni@bonollo.it

**Keywords:** neuroinflammation, 5-(3′,4′-dihydroxyphenyl)-γ-valerolactone, curcumin, synergistic effect, Nrf2 signaling, NLRP3 inflammasome

## Abstract

**Background/Objectives:** 5-(3′,4′-dihydroxyphenyl)-γ-valerolactone (γ-VL), recently identified as a predominant microbial metabolite derived from proanthocyanidins, offers benefits such as reducing inflammation, oxidative stress, and supporting brain health. Its effects on neuroinflammation and microglial activation remain largely unexplored. Curcumin, a bioactive component isolated from *Curcuma longa* L., is well known for its ability to reduce microglial activation and pro-inflammatory mediator production and release. While the individual effects of γ-VL and curcumin are well documented, their potential combined effects remain unexplored. This research sought to investigate the possible synergistic effects of γ-VL and curcumin in reducing microglial activation. **Methods:** Primary rat cortical microglia were pre-treated with γ-VL and curcumin, alone or in combination, before stimulation with LPS. An MTT assay was used to evaluate cell viability, while pro-inflammatory mediators were assessed by real-time PCR and ELISA. Nitric oxide production was evaluated with the Griess assay. SynergyFinder Plus software analyzed potential synergistic effects. **Results:** The combination of low micromolar concentrations of γ-VL and curcumin synergistically reduced LPS-induced microglial activation. Specifically, the combination exhibited a significantly greater ability to inhibit the production and release of pro-inflammatory factors (such as IL-1β, TNF-α, and NO) compared to each compound individually. Mechanistically, the anti-inflammatory activity was attributed to the downregulation of NLRP3 expression, and the reduction in microglial activation was linked to the modulation of the NOX2/Nrf2 signaling pathway. **Conclusions:** The combination of low micromolar concentrations of γ-VL and curcumin produces synergistic anti-inflammatory effects in microglia by targeting key inflammatory pathways, indicating its potential utility as a treatment strategy for neurodegenerative diseases involving microglial activation.

## 1. Introduction

Increasing life expectancy has been accompanied by a heightened incidence of neurodegenerative disorders, including Alzheimer’s and Parkinson’s diseases [1]. Despite extensive research, the exact causes and pathological mechanisms of neurodegeneration remain unclear. However, common factors include abnormal protein aggregation and deposition, mitochondrial dysfunctions, DNA damage, oxidative stress, and neuroinflammatory [2]. Inflammation localized to the central nervous system (CNS), commonly termed neuroinflammation, initiates as a protective response to injury with the aim of promoting tissue repair [3]. However, prolonged inflammation can become harmful, leading to neuronal damage and degeneration [4]. Neuroinflammation is mainly mediated by microglia and astrocytes, with microglia serving as innate immune cells of the brain. These cells serve an essential function in immune surveillance and act like macrophages, producing cytokines and chemokines [5,6]. As key immune cells within the CNS, microglia are indispensable for preserving brain health but are also implicated in neurodegenerative and neurological disorders [7]. Therefore, maintaining a proper balance between neuroprotective and pro-inflammatory microglia phenotypes could offer potential strategies for preventing or treating neuroinflammation-related diseases.

**Figure 1:**
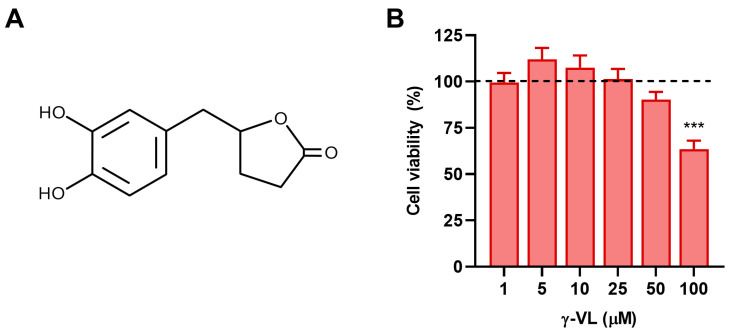
(**A**) Chemical structure of 5-(3′,4′-dihydroxyphenyl)-γ-valerolactone. (**B**) Cytotoxicity assessment in microglia cells after treatment with γ-VL at increasing concentrations. Cell viability is expressed as a percentage relative to control cells (dashed line). Data are shown as means ± SEM (*n* = 3 in triplicate) and analyzed by one-way ANOVA followed by Dunnett’s multiple comparison test. *** *p* ˂ 0.001 vs. control cells (vehicle-treated cells; dashed line).

Proanthocyanidins (PCAs), which are polyphenolic compounds originating from flavonoids, are commonly found in plant materials such as flowers, nuts, fruits, bark, and seeds [8]. PCAs exhibit several health benefits, including support for gastrointestinal functions, as well as anticancer, anti-obesity, hypoglycemic, cardiovascular protective, neuroprotective, and anti-inflammatory effects [9]. Upon reaching the colon, most ingested PCAs are metabolized by the gut microbiota into low molecular weight phenolic compounds, mainly phenyl-γ-valerolactones and phenylvaleric acids [10]. Among phenyl-γ-valerolactones, 5-(3′,4′-dihydroxyphenyl)-γ-valerolactone (γ-VL) (Figure 1A) is the main PCA metabolite produced after the consumption of flavonoid-rich diet and supplements [11,12]. It has been identified in plasma and urine, with concentrations in the low micromolar range [13] and recent research has focused on its antioxidant, anti-inflammatory, and neuroprotective activities [14,15,16]. However, the effects of γ-VL on neuroinflammation and microglia activation remain unexplored.

Unlike the limited knowledge about the effects of γ-VL on microglial cells, curcumin (diferuloylmethane), a bioactive compound found in *Curcuma longa* L. (Zingiberaceae), commonly known as turmeric, is a well-studied agent that has demonstrated efficacy in mitigating the biological processes and clinical effects associated with neuroinflammation and neurodegenerative diseases. Previously, we showed that curcumin has anti-neuroinflammatory effects, being able to reduce microglia activation both in in vitro and in vivo models [17,18,19,20,21,22].

While the anti-inflammatory activities of γ-VL and curcumin have been studied individually, their combined effects remain unexplored. Combination therapy has emerged as an effective strategy in numerous fields of intervention, including the reduction in inflammation attributed to its complex etiology and underlying pathophysiological mechanisms [23]. Combining different agents can enhance pharmacological efficacy and reduce the required doses, thereby minimizing potential side effects.

Building on this evidence, in the present investigation, we first examined the effect of γ-VL on microglia activation. Then, we explored the potential for synergistic effects of combining it with curcumin to attenuate microglial activation and neuroinflammation. Additionally, we explored the molecular mechanisms underlying the observed effects.

## 2. Materials and Methods

### 2.1. Materials

Unless otherwise specified, chemicals, antibiotics, and tissue culture media were obtained from Merck (Milan, Italy). Fetal bovine serum (FBS) was sourced from Life Technologies (San Giuliano Milanese, Italy). Lipopolysaccharide (LPS; Ultrapure LPS-EB from *Escherichia coli*, 0111:B4 strain) was purchased from InvivoGen (InvivoGen Europe, Toulouse, France). γ-VL was synthesized and kindly provided by Prof. Giancarlo Aldini (Department of Pharmaceutical Sciences, University of Milan, Milan, Italy). γ-VL and curcumin were dissolved in dimethyl sulfoxide (DMSO) and used with a final DMSO concentration of 0.1%. Endotoxin-free water (InvivoGen) was used to dissolve all water-soluble chemicals.

### 2.2. Cell Cultures

Sprague-Dawley rats (CD strain, bred in our Animal Facility) were kept in controlled temperature and humidity conditions, with ad libitum access to water and food, under a 12-h light/dark cycle. Animal care and procedures were carried out in strict adherence to the National Institutes of Health guidelines for laboratory animal care and the standards outlined by the Italian Ministry of Health (D.L. 26/2014). Formal approval to conduct the experiments described was obtained from the Animal Ethics Committee of the University of Padua (Organismo Preposto al Benessere Animale, OPBA) and authorized by the Italian Ministry of Health (Protocol Approval No. 41451.N.WBR, approved on 23 March 2023). The animal studies were conducted in compliance with the ARRIVE guidelines [24], and all measures were taken to minimize animal suffering and to reduce the number of animals involved. One-day-old rat pups of both sexes were rapidly decapitated, minimizing suffering, discomfort, and stress. The cerebral cortices were dissected, stripped of meninges, and mechanically minced with a razor blade. The minced tissue was collected and incubated for 1 h in a 37 °C water bath in L-15 medium containing 1.25 mg/mL papain (Worthington Biochemical Corporation, Lakewood, NJ, USA), 0.24 mg/mL L-cysteine, and 40 μg/mL DNase. After enzymatic digestion, the solution was removed and replaced with fresh L-15 medium containing 40 μg/mL DNase and 1 mg/mL trypsin inhibitor. After a 2-min incubation in a 37 °C water bath, the cell suspension was centrifuged at 2400 rpm for 5 min. The resulting cell pellet was resuspended in high-glucose Dulbecco’s modified eagle medium (DMEM) supplemented with 2 mM L-glutamine, 10% heat-inactivated FBS, 100 units/mL penicillin, 100 μg/mL streptomycin, and 50 μg/mL gentamicin (growth medium). The cells were then plated in T-75 flasks [25]. When the mixed glial cultures became confluent (typically 7 days post-isolation), microglia were harvested by shaking the flasks on an orbital shaker (200 rpm for 1 h at 37 °C), then re-suspended in growth medium. The suspension was transferred to Sterilin plastic Petri dishes and incubated at 37 °C for 45 min to facilitate microglial adhesion. The adherent microglial cells (>99% purity, as determined by flow cytometry using cell type-specific antibodies [26]) were mechanically detached, resuspended in growth medium, and re-plated on poly-L-lysine-coated plastic wells at a density of 1.50 × 10^5^ cells/cm^2^. Cells were maintained at 37 °C in a humidified atmosphere with 5% CO_2_/95% air.

### 2.3. Cell Treatment

Cells were seeded in poly-L-lysine-coated 48-well plates in a growth medium and allowed to adhere overnight. The serum-containing medium was then replaced with serum-free medium, and cells were pre-treated with γ-VL, curcumin, or their combination for 1 h. Next, cells were stimulated with 250 ng/mL Ultra-Pure LPS-EB (used as a positive control) for 6 or 24 h to evaluate gene expression or the secretion of pro-inflammatory mediators, respectively. Cells treated with 0.1% DMSO served as negative controls.

### 2.4. Cell Viability Assay

The viability of microglia was assessed using a colorimetric method with the metabolic dye 3-(4,5-dimethylthiazol-2-yl)-2,5-diphenyltetrazolium bromide (MTT) [27,28]. Cells, seeded in 96-well plates, were treated with γ-VL (1–100 μM) or a combination of γ-VL with curcumin for 24 h. At the end of treatments, the medium was removed, and cells were incubated with MTT (0.18 mg/mL) at 37 °C for 1 h. The supernatants were then discarded, and the purple formazan crystals formed in viable cells were solubilized with 100 μL of DMSO. To quantify the absorbance, a microplate reader was employed, setting the test wavelength to 570 nm and the reference wavelength to 630 nm. Cell viability was expressed as the percentage of viable cells compared to the control cultures treated with 0.1% DMSO.

### 2.5. Cytokine Determination

After treatment completion, culture media were collected, and interleukin (IL)-1β and tumor necrosis factor (TNF)-α levels were assessed using ELISA kits (Antigenix America, Huntington Station, NY, USA), adhering to the manufacturer’s protocol. Absorbance was measured at a wavelength of 450 nm using a microplate reader. Cytokine concentrations (pg/mL) were determined by reference to standard curves generated simultaneously.

### 2.6. Nitric Oxide Quantification Assay

The production of nitric oxide (NO) was assessed indirectly by measuring its stable oxidized products, nitrite and nitrate, using the Griess assay. An equal volume (50 µL) of culture supernatant and Griess reagent were combined. Following a 15-min incubation at room temperature, the absorbance was read at 540 nm wavelength using a microplate reader. Nitrite levels were calculated using a standard curve prepared from known concentrations of sodium nitrite.

### 2.7. Real-Time Polymerase Chain Reaction (Real-Time PCR)

The total RNA was extracted using an RNA extraction mini kit (Qiagen, Milan, Italy). Complementary DNA synthesis was carried out with Superscript IV reverse transcriptase (Thermo Fischer Scientific, Waltham, MA, USA). The real-time PCR reaction was performed using the SYBR Green JumpStart Taq ReadyMix on an AriaMX thermal cycler (Agilent Technologies, Santa Clara, CA, USA). Thermal cycling involves an initial denaturation at 94 °C for 4 min, followed by 40 cycles of 94 °C for 20 s, 60 °C for 15 s, and 72 °C for 30 s. Primer sequences are provided in Table 1. Gene expression levels were determined by linear regression analysis using standard curves, with amplification efficiencies between 90 and 100%. Dissociation curves of each primer pair demonstrated the amplification of a single product. Data were normalized to β-actin mRNA levels and results were presented as fold changes relative to the control group (vehicle-treated cells).

### 2.8. Synergistic Effect Analysis

The evaluation of the effect of the combination of curcumin and γ-VL on the production of pro-inflammatory mediators was performed by means of SynergyFinder Plus 07.09.2024-R-3.10.3, a software based on the “R” package SynergyFinder R package 3.10.3 (https://bioconductor.org/packages/release/bioc/html/synergyfinder.html (accessed on 7 March 2025)), available as a web resource (https://www.synergyfinderplus.org/ (accessed on 7 March 2025); Netphar, Faculty of Medicine, University of Helsinki) [29]. The program allows the estimation of the drug interaction expressed as synergy scores according to four main theoretical models (Highest Single Agent: HSA; Bliss; Loewe; Zero Interaction Potency: ZIP), together with drug interaction graphs in two- and three-dimensional visualization. A comprehensive discussion of the theoretical frameworks for analyzing drug combinations is available in [30]. Briefly, the models can be classified as effect-based and dose-effect-based. The effect-based approaches include HSA and Bliss models, which assess the response of positive interactions by examining each compound in a combination individually. The dose-effect-based approaches comprise Loewe and ZIP models, which consider the dose of each compound producing the same effect in the considered pharmacological system. The summary synergy score obtained with the theoretical analysis of experimental data can be understood as the average additional response attributed to drug interactions (for instance, a synergy score of 10 indicates a 10% increase in response beyond what was anticipated). A synergy score close to 0 suggests low confidence for synergism or antagonism. Synergy scores less than −10 suggest antagonistic interaction and a score in the interval of −10 to 10 is likely to be additive. Conversely, a synergy score greater than 10 indicates a synergistic interaction (https://synergyfinder.fimm.fi (accessed on 7 March 2025)). The software also provides the significance (*p*-value) for the estimated average synergy score versus the score of zero under the null hypothesis of non-interaction [29]. The procedure was performed using SynergyFinder Plus with no baseline correction mode, since the data were already normalized to baseline.

Each model may have strengths and limitations, so in the present research, all four models were considered, in order to compare their outputs to obtain a robust understanding of the drug interaction landscape. The ZIP model appears to have overcome several limitations of the other existing models [31] and, for this reason, was selected in our study as the main reference method. The other models have been evaluated as well since they differ in their null hypotheses of non-interaction, and therefore the synergy may not always align with one another [29]. While the four synergy models are widely used to assess drug synergy, specific applications to anti-inflammatory drugs are less commonly detailed in the literature. However, the algorithm remains valid in analyzing pre-clinical drug combination datasets [29]. For instance, Zimmermann et al. [32] and Caesar and Cech [33] considered synergy investigations in natural product extracts.

Also, the Combination Index (CI) was obtained with the routines of the above-mentioned software. The CI is related to Loewe’s additivity model as well as to isobologram [34] and has been further improved by Chou and Talalay [35,36]. A CI of 1 signifies additivity; a CI value below 1 suggests synergism, whereas a value above 1 indicates antagonism. In the literature, synergistic anti-inflammatory mechanisms have been investigated for natural anti-inflammatory compounds using the Chou-Talalay method for CI [37].

### 2.9. Statistical Analysis

All data are based on at least three separate experiments that were run in triplicate. The mean ± standard error of the mean (SEM) is used to express the results. The statistical analysis was conducted using GraphPad Prism Software, version 8.4 (San Diego, CA, USA). As explained in the figure legends, one-way ANOVA was used to compare the groups, and post hoc tests were then conducted. To control the family-wise error rate when conducting multiple comparisons, the Holm–Sidak test was used. Dunnett’s multiple comparison test was used to compare multiple treatment groups with a control mean. Differences were considered statistically significant when *p* < 0.05.

## 3. Results

### 3.1. Evaluation of Non-Cytotoxic Concentrations of γ-VL in Microglial Cells

Initially, we conducted experiments to assess the cytotoxicity and determine non-cytotoxic concentrations of γ-VL in microglia cells. Cultures were incubated with increasing concentrations of γ-VL (1–100 µM) for 24 h, and cell viability was determined by the MTT assay. As presented in Figure 1B, no toxic effects were observed at γ-VL concentrations ranging from 1 to 50 μM, whereas 100 μM γ-VL caused a significant reduction in microglia viability. Based on these results, concentrations of γ-VL in the range of 1 to 50 µM were selected for subsequent studies.

### 3.2. γ-VL and Curcumin Reduced the Release of Pro-Inflammatory Factors from LPS-Stimulated Microglial Cells

**Figure 2:**
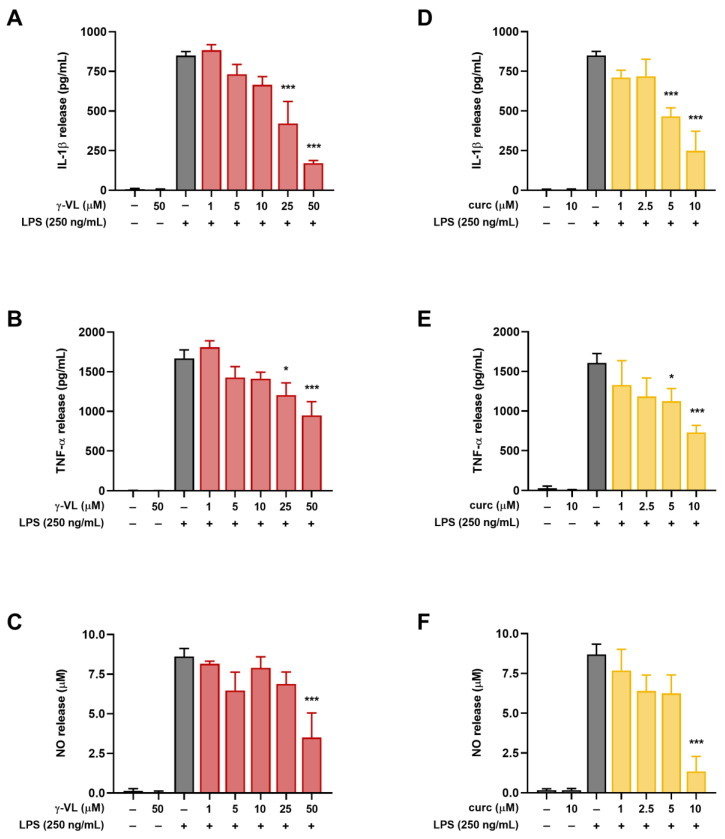
Effect of γ-VL and curcumin on pro-inflammatory mediator release from primary cortical microglia. Concentration-dependent response of (**A**–**C**) γ-VL and (**D**–**F**) curcumin on LPS-induced secretion of IL-1β, TNF-α, and NO from microglia. Data are expressed as the mean ± SEM from 4 independent experiments, each performed in triplicate. Data were analyzed by one-way ANOVA followed by Holm–Sidak’s multiple comparison test. * *p* < 0.05 and *** *p* < 0.001 compared to LPS-treated cells (gray bars).

IL-1β, TNF-α and NO are some of the most important cellular mediators secreted in response to LPS. Therefore, to investigate whether γ-VL modulates the release of these pro-inflammatory molecules, primary microglia cells were pre-treated for 1 h with γ-VL at concentrations that do not affect microglia viability (1–50 μM). Subsequently, the cells were exposed to LPS (250 ng/mL; positive control cells) for 24 h to induce an inflammatory response. As shown in Figure 2A–C, γ-VL reduced the increased levels of pro-inflammatory mediators released into the culture medium following LPS stimulation. Specifically, γ-VL significantly reduced the release of IL-1β and TNF-α starting from the concentration of 25 µM (Figure 2A,B), whereas NO release was reduced at a concentration of 50 µM (Figure 2C). Of note, γ-VL itself, at the highest concentration tested (i.e., 50 µM), did not affect the low levels of the pro-inflammatory mediators released by unstimulated microglia (second bars in Figure 2A–C).

Consistent with our previous studies [17,18,20,22], non-cytotoxic concentrations of curcumin (1–10 µM) significantly suppressed LPS-induced microglial activation also under the experimental conditions used here (Figure 2D–F).

### 3.3. The Combined Treatment with γ-VL and Curcumin Exhibited a Synergistic Inhibition of the Pro-Inflammatory Response in Microglia

**Figure 3:**
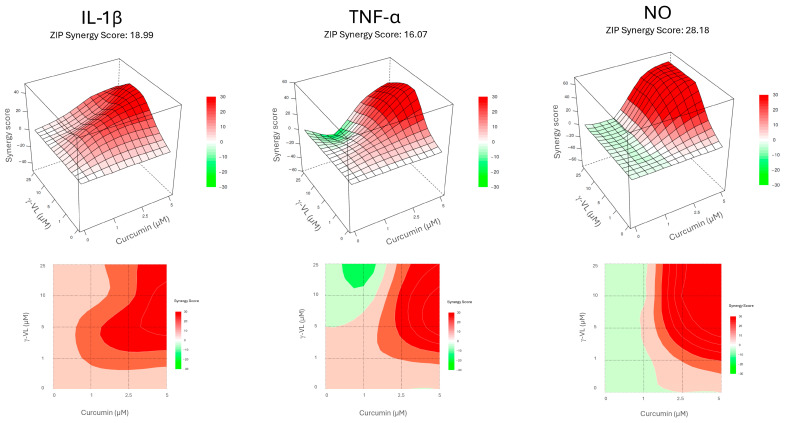
Concentration–response maps depicting the inhibition of the pro-inflammatory mediator release obtained with the tested γ-VL and curcumin combinations, according to ZIP model. The upper panel shows 3D surface, while the lower panel shows 2D contour. For each mediator, the average synergy score is indicated. Red color indicates synergism, while green color suggests antagonistic interaction.

**Figure 4:**
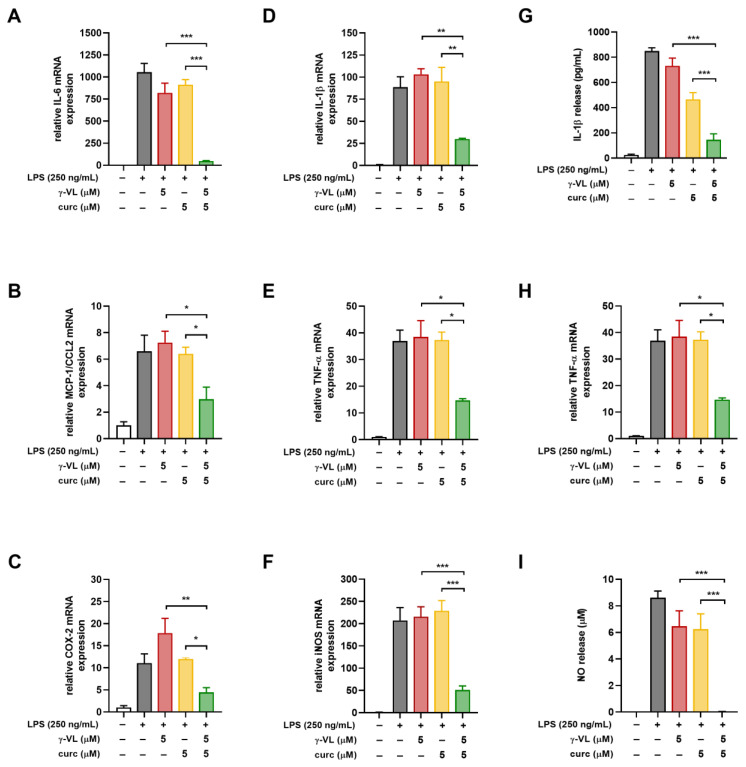
Effect of the combination of γ-VL and curcumin on pro-inflammatory mediator production and release from cortical microglia. (**A**–**F**) mRNA levels of inflammatory factors including IL-6, MCP-1/CCL2, COX-2, IL-1β, TNF-α, and iNOS were tested by real-time PCR. (**G**–**I**) Levels of IL-1β, TNF-α, and NO released into the medium were assessed using ELISA and the Griess reaction. Data are presented as mean ± SEM (*n* = 3 in triplicate) and analyzed by one-way ANOVA followed by Holm–Sidak’s test. * *p* ˂ 0.05, ** *p* ˂ 0.01, and *** *p* ˂ 0.001. Vehicle-treated cells, white bars; LPS-treated cells, gray bars; LPS + γ-VL-treated cells, red bars; LPS + curcumin-treated cells, yellow bars; LPS + γ-VL + curcumin-treated cells, green bars.

Before evaluating the anti-inflammatory effect of γ-VL (1–25 µM) combined with curcumin (1–5 µM), we assessed the potential cytotoxicity of the combination using the MTT assay. The combination, at different concentrations, did not significantly alter microglia cell viability (Figure S1). The absence of a significant effect on microglial viability suggests that the combination treatment is well tolerated by these cells, supporting its potential for further investigation. The combination of γ-VL and curcumin was then evaluated for potential additive or synergistic anti-inflammatory effects by using the previously assessed non-cytotoxic concentrations of the two compounds, which corresponded to low, medium, and high concentrations, also considering possible clinically relevant concentrations. By using the four theoretical models for drug combination screening offered by SynergyFinder Plus, it was possible to observe a confident synergistic effect of γ-VL and curcumin. Considering for instance the ZIP model, which permits an evaluation of interactions in a systematic and quantitative manner, the mean synergy score for the inhibition of IL-1β was 18.99, for TNF-α 16.07, and for NO 28.18 (*p* < 0.0001 against the null hypothesis of non-interaction) (Figure 3). The mean values for all the three pro-inflammatory mediators exceeded the conventional value of 10, probative of a synergistic interaction between the two agents. Table S1 also presents detailed synergy scores for all the tested combinations of γ-VL and curcumin, confirming the entity of synergism for most of the combinations, although with some concentrations, being the score in the range −10 to 10, an additive effect emerges. The other three tested models confirmed the results of the combination (Figures S2–S4, and Table S1). The agreement between the HSA, Bliss, and Loewe models with the ZIP model strengthens the reliability of the combination effect assessment, highlighting the robustness of the findings. The concordance of the models suggests consistent combinatory interaction, reinforcing the observed effects and minimizing the likelihood of model-specific bias. Moreover, the calculated CI (Table S1), which was nearly always less than 1, confirmed the synergism of the combination of γ-VL and curcumin in reducing the release of the three pro-inflammatory mediators. In two combinations only the CI was close to or exceeding 1, likely due to variability of data. To illustrate the behavior of the two compounds in terms of absolute values, Figure 4 shows that the combination of 5 µM γ-VL and 5 µM curcumin (green bars) exerted a stronger inhibitory effect on LPS-induced expression of mRNA for several pro-inflammatory factors, including IL-6, monocyte chemoattractant protein-1 (MCP-1/CCL2), COX-2, IL-1β, TNF-α, and inducible nitric oxide synthase (iNOS), as well as on the release of IL-1β, TNF-α, and NO compared to γ-VL (red bars) and curcumin (yellow bars) alone. Given the robust synergistic effect observed with this combination, also confirmed by the analysis through the four synergy models, we selected these concentrations for subsequent mechanistic studies.

### 3.4. The Combination of γ-VL and Curcumin Synergistically Reduced NLRP3 Expression and Restored the NOX2/Nrf2 Balance

**Figure 5:**
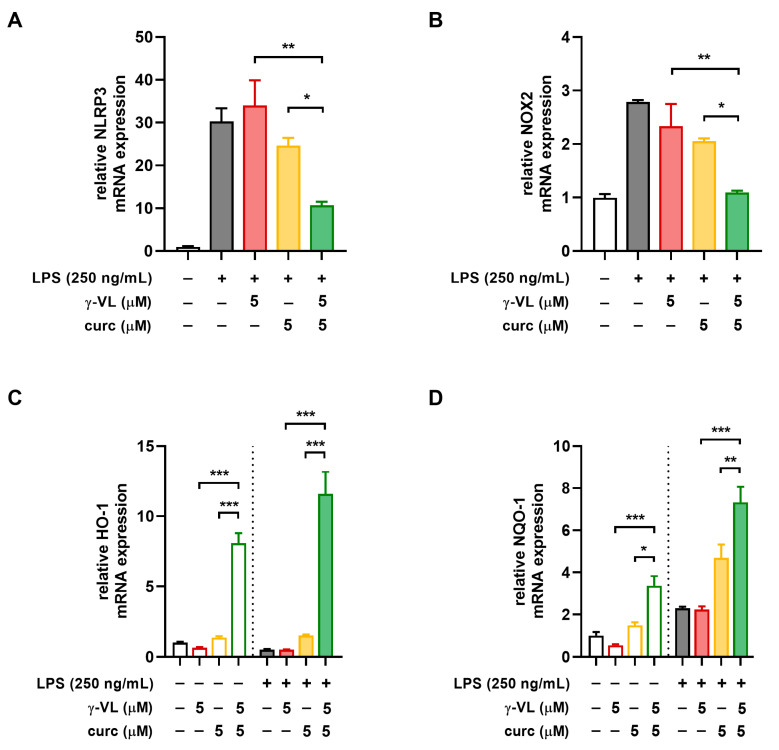
Effect of the combination of γ-VL and curcumin on NLRP3 expression and NOX2/Nrf2 balance in primary microglia. Real-time PCR analysis of the mRNA expression of (**A**) NLRP3, (**B**) NOX2, (**C**) HO-1, and (**D**) NQO-1. Data are presented as means ± SEM (*n*= 3 in triplicate) and analyzed using one-way ANOVA with Holm–Sidak’s test for post hoc comparisons. * *p* ˂ 0.05, ** *p* ˂ 0.01, and *** *p* ˂ 0.001. Vehicle-treated cells, white bars; γ-VL treatment, red hollow bars; curcumin treatment, yellow hollow bars; γ-VL + curcumin treatment, green hollow bars; LPS-treated cells, gray-filled bars; LPS + γ-VL treatment, red-filled bars; LPS + curcumin treatment, yellow-filled bars; LPS + γ-VL + curcumin-treatment, green-filled bars.

The NLRP3 inflammasome is a protein complex consisting of the innate immune receptor protein NLRP3, the adapter protein ASC, and the inflammatory protease caspase-1. Its activation induces the synthesis and release of mature IL-1β and IL-18, thereby driving inflammatory responses [38]. Accordingly, we evaluated the effect of γ-VL, curcumin, and their combination on NLRP3 mRNA expression levels in LPS-treated microglia. Treatment with the combination of γ-VL (5 μM) and curcumin (5 μM) resulted in a significant reduction in LPS-induced NLRP3 overexpression (green bar in Figure 5A), an effect not observed when the two compounds were used individually at the same concentrations (red and yellow bars in Figure 5A). These results indicate that the NLRP3 inflammasome is a key target for the anti-inflammatory effects exerted by γ-VL and curcumin in combination.

Oxidative stress, caused by altered production of reactive oxygen species (ROS) is a common feature characteristic of both neuroinflammation and neurodegenerative diseases [39]. NADPH oxidases (NOX) are the primary sources of ROS in immune cells. Specifically, NOX2 is constitutively expressed in microglia, where it is essential for regulating inflammatory and immunological responses as well as for promoting the generation of ROS [40]. While NOX2 promotes oxidative stress, nuclear factor erythroid 2-related factor 2 (Nrf2) counteracts it by inducing the expression of antioxidant and neuroprotective genes [41,42]. In microglia, the combination of γ-VL and curcumin significantly reduced the mRNA expression of NOX2 (Figure 5B) while enhancing the expression of two key Nrf2 target genes, such as heme-oxygenase 1 (HO-1) and NAD(P)H quinone dehydrogenase 1 (NQO-1). Notably, these effects were substantially more pronounced than those observed with individual treatments (Figure 5C,D).

## 4. Discussion

PACs are secondary metabolites widely distributed in plants and known for their various beneficial effects. Extensive research has highlighted their positive effects on the gastrointestinal tract, as well as their anti-obesity, anti-carcinogenic, anti-diabetic, anti-inflammatory, and neuroprotective properties [43,44,45]. When ingested, a significant proportion of PACs is metabolized by the gut microbiota into compounds with bioavailability and biological activity, which can influence various physiological processes. Among the metabolites produced, phenolic acids and phenyl-γ-valerolactones have been detected in human urine and blood samples [46]. A recent study investigated Ecovitis^®^, a highly standardized grape seed extract, developed by Distillerie Bonollo Umberto S.p.A., which is enriched with polymeric PACs. The study identified γ-VL as the key component responsible for the anti-inflammatory and antioxidant effects of PACs [16]. Moreover, γ-VL has been shown to exhibit anti-amyloid and neuroprotective effects, preventing memory impairment in animal models of Alzheimer’s disease [14,47]. However, its impact on neuroinflammation and microglial activation is still not well established. In this investigation, we show for the first time that γ-VL, at concentrations of 25 and 50 µM, reduced the LPS-induced microglial activation in vitro. This effect was linked to the suppression of IL-1β and TNF-α release, two key pro-inflammatory cytokines that are among the first to be produced during inflammation and have been implicated in neurodegeneration due to their sustained levels [48]. Additionally, γ-VL showed a significant effect on the microglial inflammatory response also by reducing the production of NO induced by LPS. However, the effects of γ-VL were observed only at high concentrations (i.e., 25 and 50 µM), which may be associated with side effects. To overcome this limitation, several studies have shown that combining natural compounds enhances therapeutic outcomes in various in vitro and in vivo models. For example, combinations of polyphenols have shown greater effectiveness than single polyphenol treatments as anti-cancer agents and in conditions related to oxidative stress and inflammation [49]. Based on this evidence, we tested the anti-neuroinflammatory effects γ-VL in combination with curcumin, a broadly active natural compound with multiple molecular targets and diverse mechanisms of action, including the ability to suppress the microglia release of inflammatory cytokines, as shown in our previous studies [17,18,19,20,21,22]. Our results clearly show that γ-VL and curcumin, in a low micromolar range, synergistically inhibited LPS-induced inflammation in microglial cells, as evidenced by reduced expression of various pro-inflammatory genes and lower secretion of IL-1β, TNF-α, and NO in the culture medium. These results are of notable importance because, although the anti-inflammatory effects of γ-VL and curcumin are already known, their combined effects have never been investigated, especially in a model of neuroinflammation. Furthermore, it is important to emphasize that our findings are highly relevant, especially considering the poor absorption, rapid metabolism, and fast systemic clearance of curcumin, factors that result in low plasma and tissue levels, particularly in the CNS, thereby limiting therapeutic use of curcumin [50]. To improve bioavailability and blood–brain barrier permeation, nanoformulations combining γ-VL and curcumin could offer a promising approach to addressing chronic neuroinflammation.

Drug combinations are crucial in the management of various pathologies and in the study of new molecules with potential medical applications. The synergistic combination of drugs can produce greater effects than the simple sum of each component (where only additivity occurs), producing the so-called “1 + 1 > 2”, effect [51] that allows for dose reduction, potentially leading to a better tolerance profile while maintaining the same overall efficacy [52]. The availability of updated informatics tools allowed us to investigate the specific interaction between the two agents γ-VL and curcumin. In particular, the summary synergy score, determined through four of the major models of drug interaction (HSA, Bliss, Loewe, and ZIP), permitted to ascertain, across a range of concentrations, the presence of a supra-additive interaction, i.e., true synergism [53,54], of γ-VL and curcumin combination on inhibiting the release of three pro-inflammatory mediators (IL-1β, TNF-α, and NO). Since the four models offered by SynergyFinder R package-based software do not have the same null hypotheses of non-interaction [29], their results may not be fully superimposable. The strict similitude among the results obtained here with all the models strongly supports the presence of the synergistic interaction between the two compounds in the anti-inflammatory mechanism. Moreover, interaction can be considered of high entity, since the summary synergy score exceeded for all models the values of 10, considered suggestive of a synergistic interaction. Also, by using another measure of drug interaction, the CI, which is related to classical isobologram analysis [34,36], the occurrence of synergism was confirmed for γ-VL and curcumin combination.

The molecular mechanisms that cause inflammation have been investigated, and various enzymes, chemokines, cytokines, and transcription factors that can mediate inflammation have been identified. The NLRP3 inflammasome is a protein complex involved in the regulation of innate immunity by activating caspase-1 and providing a platform for the synthesis of IL-1β and IL-18, which consequently amplifies inflammation [55,56]. Given its involvement in numerous pathological conditions, the NLRP3 inflammasome has become a therapeutic target of great interest and modulating its activity could offer new strategies for the treatment of inflammatory diseases. In microglia cells, pre-treatment with the combination of γ-VL and curcumin significantly reduced NLRP3 overexpression induced by LPS. This suggests that the modulation of NLRP3 expression, which is a critical checkpoint for NLRP3 inflammasome activation [57], could represent a major anti-inflammatory mechanism of the combination. Notably, the effect on NLRP3 expression was not observed when γ-VL and curcumin were used individually at the same concentration used in the combination, confirming the synergistic effect.

Oxidative stress, associated with the overproduction of reactive species alongside diminished antioxidant protection, plays a significant role in the onset and progression of inflammation and neurodegenerative diseases. Microglia constitutively express the NOX2 enzyme complex, which contributes to neuroinflammation by producing ROS that can directly damage surrounding cells. This damage involves the peroxidation of lipids, proteins, and nucleic acids, as well as the disturbance of subcellular organelles, and the initiation of abnormal physiological functions. These events are hallmark features of oxidative stress and further amplify ROS generation. Additionally, ROS produced by NOX2 activates different nuclear transcription factors, thereby enhancing the expressions of pro-inflammatory mediator genes [58]. Conversely, Nrf2 signaling is essential for protecting against oxidative stress and maintaining cellular and tissue homeostasis, as well as redox balance [59]. Nrf2 is responsible for regulating antioxidant gene expression, thereby exerting anti-inflammatory effects, including glutathione S-transferases, NQO-1, and HO-1, among others. Consequently, stimulating Nrf2 signaling appears to be a promising strategy for reducing neuroinflammation and neurodegeneration. For instance, curcumin exhibits anti-inflammatory properties by activating Nrf2 and up-regulating HO-1 expression [60] and γ-VL can bind to the cysteine residues of Keap1, the negative regulator of Nrf2, thereby activating the Nrf2 pathway [16]. In our experimental conditions, the combination of γ-VL and curcumin in a low micromolar range reduced LPS-induced NOX2 overexpression while increasing the expression of two downstream genes of Nrf2 signaling, HO-1 and NQO-1. As previously observed for NLRP3 expression, these effects were detected only with the drug combination, while the two molecules alone at the concentration of 5 µM did not modify the expression of the analyzed genes. Taken together, these findings suggest that the modulation of NOX2 expression, which promotes oxidative stress, and the Nrf2 signaling pathway, which enhances antioxidant defenses, may contribute to the anti-inflammatory properties of γ-VL and curcumin combination. Indeed, by balancing NOX2/Nrf2 activity, the combination of γ-VL and curcumin markedly reduced LPS-induced NLRP3 overexpression, a key driver of the inflammatory response. Numerous studies have shown that the transcription factor NF-κB is involved in the overexpression and activation of NLRP3, with the NF-κB/NLRP3 axis playing a critical role in neuroinflammation. In fact, both curcumin and γ-VL have been shown to inhibit the NF-κB signaling pathway [16,18], and this may also underlie the mechanism of action of their combination.

In the present study, we demonstrated, for the first time, that the synergistic effect of the γ-VL and curcumin combination represents a significant achievement. This combination could reduce the required doses of each compound to achieve the anti-inflammatory effect while simultaneously minimizing the risk of adverse events associated with higher doses of the individual compounds. This aspect is particularly relevant for molecules with antioxidant activity, such as those studied here, as certain antioxidants (e.g., vitamins and flavonoids) can exhibit pro-oxidant activity and promote the synthesis of pro-inflammatory molecules when used at high doses [61,62].

## 5. Conclusions

**Figure 6:**
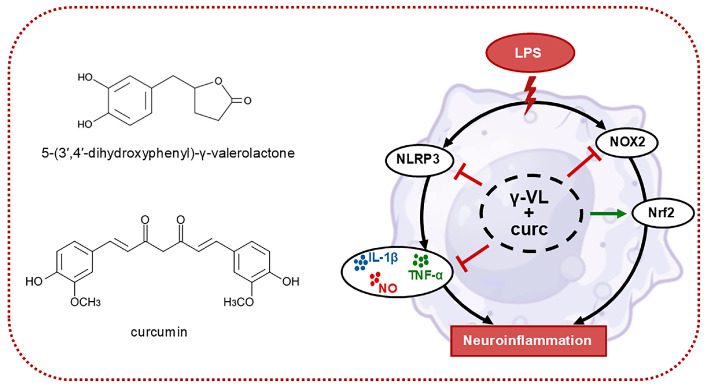
Schematic representation of the mechanism driving the anti-neuroinflammatory effects of the γ-VL and curcumin combination. Blunt arrows (┴) indicate inhibition; the sharp arrow (→) indicates stimulation.

The combination of γ-VL and curcumin in a low micromolar range exhibited synergistic anti-inflammatory effects by significantly decreasing the production and release of pro-inflammatory mediators at lower concentrations compared to the individual compounds. The synergistic effect was attained by modulating critical inflammatory and oxidative pathways. Specifically, the combination of γ-VL and curcumin synergistically downregulated the expression of NLRP3 and NOX2, a significant contributor to ROS production in microglial cells. Furthermore, it upregulated the expression of Nrf2 target genes, including NQO-1 and HO-1, which contribute to enhancing antioxidant defenses (Figure 6).

We acknowledge certain limitations of this study, particularly the use of an in vitro approach, which may not fully recapitulate the complex cell interactions and the pharmacokinetic processes occurring in vivo. However, the results of our study highlight the potential of the combination of γ-VL and curcumin as a pharmacological strategy for neurodegenerative diseases associated with microglial activation and neuroinflammation. These findings offer promising evidence to justify future in vivo research to assess the efficacy of this combination as a prophylactic and/or therapeutic intervention.

## Figures and Tables

**Table 1:** Primer sequences used in the current study.

Gene Target	Forward Primer (5′-3′)	Reverse Primer (5′-3′)
β-actin	GATCAGCAAGCAGGAGTACGATGA	GGTGTAAAACGCAGCTCAGTAACA
COX-2	TTTCAATGTGCAAGACCCGC	ACAGCTCAGTTGAACGCCTT
HO-1	GTTTCCTGTTGGCGACCGTG	GCCAGGCAAGATTCTCCCCT
IL-1β	CGTCCTCTGTGACTCGTGGG	ATGGGTCAGACAGCACGAGG
IL-6	AGAGTCACAGAAGGAGTGGCTA	CTTAGGCATAGCACACTAGGT
iNOS	GGGAACACCTGGGGATTTTC	CACAGTTTGGTCTGGCGAAG
MCP-1/CCL2	GAGATCTGTGCTGACCCCAA	TGAAGTCCTTAGGGTTGATGCA
NLRP3	TGATGCATGCACGTCTAATCTC	CAAATCGAGATGCGGGAGAG
NOX2	ATCACATCCTCCACCAAAACCATT	GCAAGGCCGATGAAGAAGATCA
NQO-1	AACGAGGTCAGATTAGGGGC	AGAGTATTTTCCCCGCTCGC
TNF-α	GCAGGTTCCGTCCCTCTCAT	TGCCAGTTCCACATCTCGGA

## Data Availability

The original contributions presented in this study are included in the article/Supplementary Material. Further inquiries can be directed to the corresponding author, who will provide the data on an individual basis to ensure that it is used in the appropriate context and with a proper understanding of the methodologies and conditions under which it was collected.

## Supplementary Materials

The following supporting information can be downloaded at https://www.mdpi.com/article/10.3390/nu17081316/s1: Figure S1: Cytotoxicity assessment in microglia cells after treatment with the combination of γ-VL and curcumin at different concentrations; Figure S2: Concentration–response maps of pro-inflammatory mediator inhibition obtained with the tested curcumin and γ-VL combinations, according to the Loewe model; Figure S3: Concentration–response maps of pro-inflammatory mediator inhibition obtained with the tested curcumin and γ-VL combinations, according to the Bliss model; Figure S4: Concentration–response maps of pro-inflammatory mediator inhibition obtained with the tested curcumin and γ-VL combinations, according to the HSA model; Table S1: Synergy scores according to the four models and CI obtained for all the tested combinations of drugs.

## Abbreviations

The following abbreviations are used in this manuscript:

CI	Combination Index
CNS	Central nervous system
DMEM	Dulbecco’s modified eagle medium
DMSO	Dimethyl sulfoxide
FBS	Fetal bovine serum
HO-1	Heme-oxygenase 1
HSA	Highest single agent
IL	Interleukin
iNOS	Inducible nitric oxide synthase
LPS	Lipopolysaccharide
MTT	3-(4,5-dimethylthiazol-2-yl)-2,5-diphenyltetrazolium bromide
NO	Nitric oxide
NQO-1	NAD(P)H quinone dehydrogenase
Nrf2	Nuclear factor erythroid 2-related factor 2
PCAs	Proanthocyanidins
ROS	Reactive oxygen species
SEM	Standard error of the mean
TNF	Tumor necrosis factor
ZIP	Zero interaction potency
γ-VL	5-(3′,4′-dihydroxyphenyl)-γ-valerolactone
