# Supplementary material for: A Combination of 5-(3′,4′-Dihydroxyphenyl)-γ-Valerolactone and Curcumin Synergistically Reduces Neuroinflammation in Cortical Microglia by Targeting the NLRP3 Inflammasome and the NOX2/Nrf2 Signaling Pathway
Source: Nutrients. 2025 Apr 10;17(8):1316. doi: 10.3390/nu17081316 (PMC12030566; doi:10.3390/nu17081316)
Supplement: Supplementary file 1 [file nutrients-17-01316-s001.zip › nutrients-3545273-supplementary.pdf]

nutrients

MDPI

Type of the Paper: Article

A combination of 5-(3′,4′-dihydroxyphenyl)-γ-valerolactone and curcumin synergistically reduces neuroinflammation in cortical microglia by targeting the NLRP3 inflammasome and the NOX2/Nrf2 signaling pathway

Emma Marcolin [1], Chiara Chemello [1], Anna Piovan [1], Massimo Barbierato [1], Paolo Morazzoni [2], Eugenio Ragazzi [1], Morena Zusso [1,*]

1 Department of Pharmaceutical and Pharmacological Sciences, University of Padua, Padua, Italy
2 Nutraceutical Division, Distillerie Umberto Bonollo S.p.A., Mestrino (PD), Italy
* Correspondence: morena.zusso@unipd.it; Tel.: +39 049 8275088

Supplementary Materials

Table of Contents

Table S1 2
Figure S1 4
Figure S2 4
Figure S3 5
Figure S4 5

Academic Editor: Firstname Lastname

Received: date
Revised: date
Accepted: date
Published: date

Citation: To be added by editorial staff during production.

Copyright: © 2025 by the authors. Submitted for possible open access publication under the terms and conditions of the Creative Commons Attribution (CC BY) license (https://creativecommons.org/licenses/by/4.0/).

Table S1. Synergy scores according to the four models, and CI obtained for all the tested combinations of drugs.

Pro-inflammatory mediator	Curcumin [μM]	γ-VL [μM]	ZIP synergy score	HSA synergy score	Loewe synergy score	Bliss synergy score	CI	Response %
IL-1β	1	1	12.73	*5.19*	*8.60*	8.57	0.43	21.51
IL-1β	2.5	1	16.73	23.55	15.87	26.97	0.55	38.91
IL-1β	5	1	14.37	12.82	14.69	15.04	0.6	57.99
IL-1β	1	5	17.16	23.16	16.44	11.57	0.22	39.48
IL-1β	2.5	5	27.76	46.83	29.02	35.11	0.26	62.19
IL-1β	5	5	34.35	37.67	29.42	30.08	0.22	82.84
IL-1β	1	10	*5.11*	10.64	*-0.88*	*-2.15*	0.28	32.29
IL-1β	2.5	10	16.41	26.76	*5.11*	14.73	0.41	48.41
IL-1β	5	10	36.43	49.88	36.56	38.01	0.08	95.05
IL-1β	1	25	*5.94*	11.14	*8.10*	*3.04*	0.11	61.52
IL-1β	2.5	25	18.58	32.01	18.84	24.39	0.12	82.39
IL-1β	5	25	22.35	42.84	19.54	20.43	0.1	93.22
TNF-α	1	1	*7.04*	*3.32*	*1.85*	10.76	0.74	24.54
TNF-α	2.5	1	10.65	*8.60*	*7.51*	15.05	0.27	40.32
TNF-α	5	1	13.87	11.70	11.56	18.00	0.32	45.04
TNF-α	1	5	*3.73*	12.49	*7.32*	*4.29*	0.23	33.71
TNF-α	2.5	5	23.68	27.53	25.67	20.42	0.04	59.25
TNF-α	5	5	48.02	52.70	52.38	45.76	0	86.04
TNF-α	1	10	*-7.30*	*6.71*	*-0.93*	*-4.71*	0.47	27.93
TNF-α	2.5	10	23.37	36.61	34.64	26.71	0.02	68.33

 https://doi.org/10.3390/nu17081316

Pro-inflammatory mediator	Curcumin [μM]	γ-VL [μM]	ZIP synergy score	HSA synergy score	Loewe synergy score	Bliss synergy score	CI	Response %
TNF-α	5	10	43.54	57.48	57.12	47.81	0	90.82
TNF-α	1	25	*-18.65*	*-13.21*	*-15.60*	*-28.11*	2.74	16.56
TNF-α	2.5	25	14.65	33.63	31.63	13.30	0.02	65.35
TNF-α	5	25	30.23	46.90	46.51	27.06	0.01	80.24
NO	1	1	*-2.17*	*2.60*	*-4.27*	*-2.17*	1.03	13.36
NO	2.5	1	11.03	15.43	14.18	11.46	0.23	41.24
NO	5	1	20.73	24.61	24.52	20.73	0.22	52.12
NO	1	5	*0.85*	*5.53*	*2.84*	*-2.55*	0.25	30.44
NO	2.5	5	37.56	48.73	46.88	30.25	0.08	74.54
NO	5	5	59.55	72.49	72.31	54.43	0	100
NO	1	10	*-3.04*	11.90	*4.75*	*4.40*	0.48	22.66
NO	2.5	10	51.39	69.12	67.23	62.89	0.12	94.93
NO	5	10	59.59	72.49	72.30	66.40	0	100
NO	1	25	*-4.06*	*0.49*	*2.70*	*-8.11*	0.74	20.61
NO	2.5	25	47.18	57.71	55.81	42.78	0.31	83.52
NO	5	25	59.60	72.49	72.29	57.91	0	100

Synergy scores lower than 10 are shown in italics.

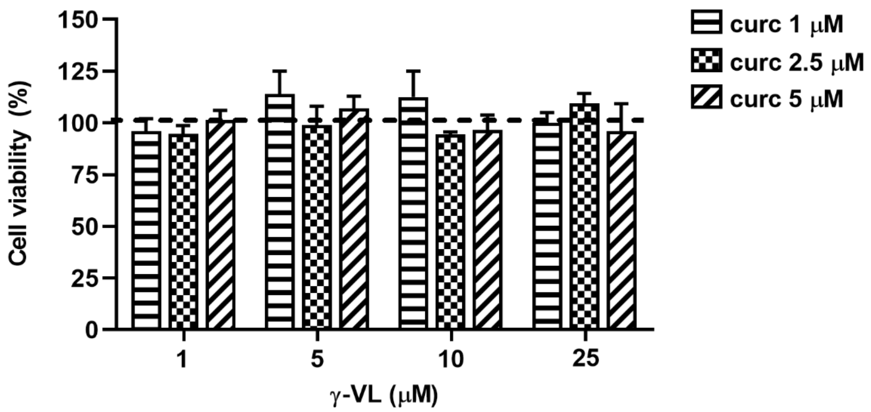

Figure S1. Cytotoxicity assessment in microglia cells after treatment with the combination of γ-VL and curcumin at different concentrations. Results are expressed as the percentage of cell viability relative to control cells (dashed line) and are shown as mean ± SEM (n = 3, performed in triplicate).

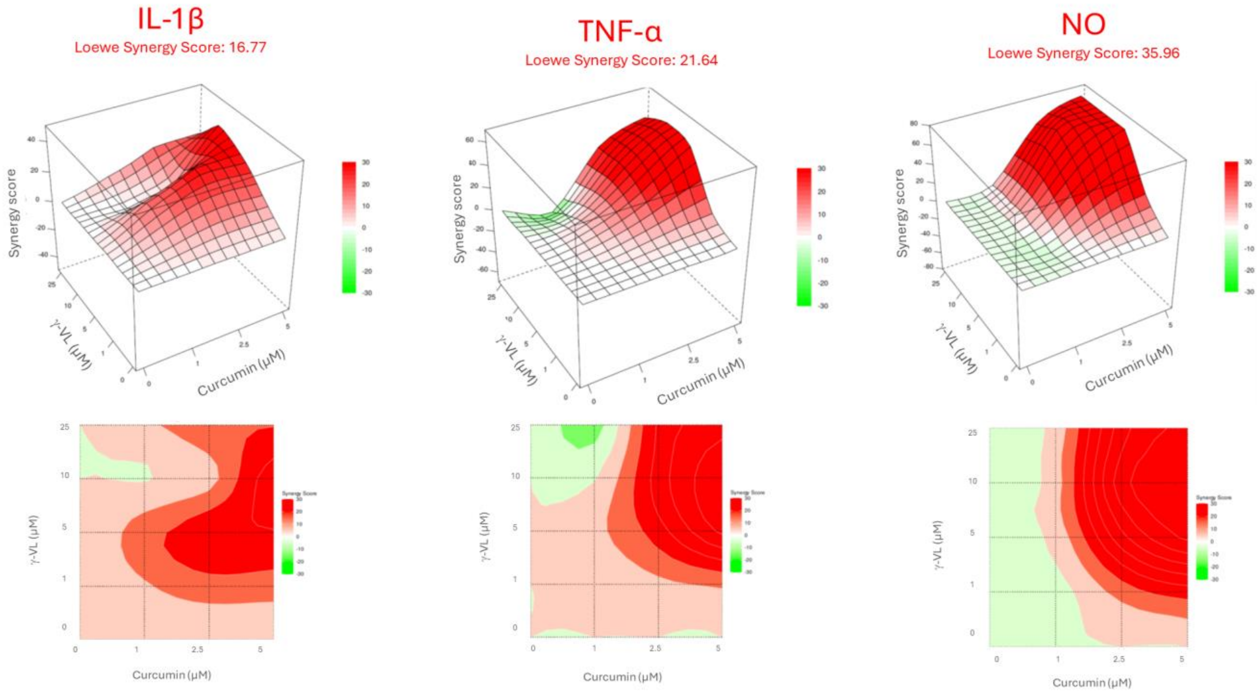

Figure S2. Concentration–response maps of pro-inflammatory mediator inhibition obtained with the tested curcumin and γ-VL combinations, according to Loewe model. The upper panel shows 3D surface, while the lower panel shows 2D contour. For each mediator, the average synergy score is indicated. Red color indicates synergism, while green color suggests antagonistic interaction.

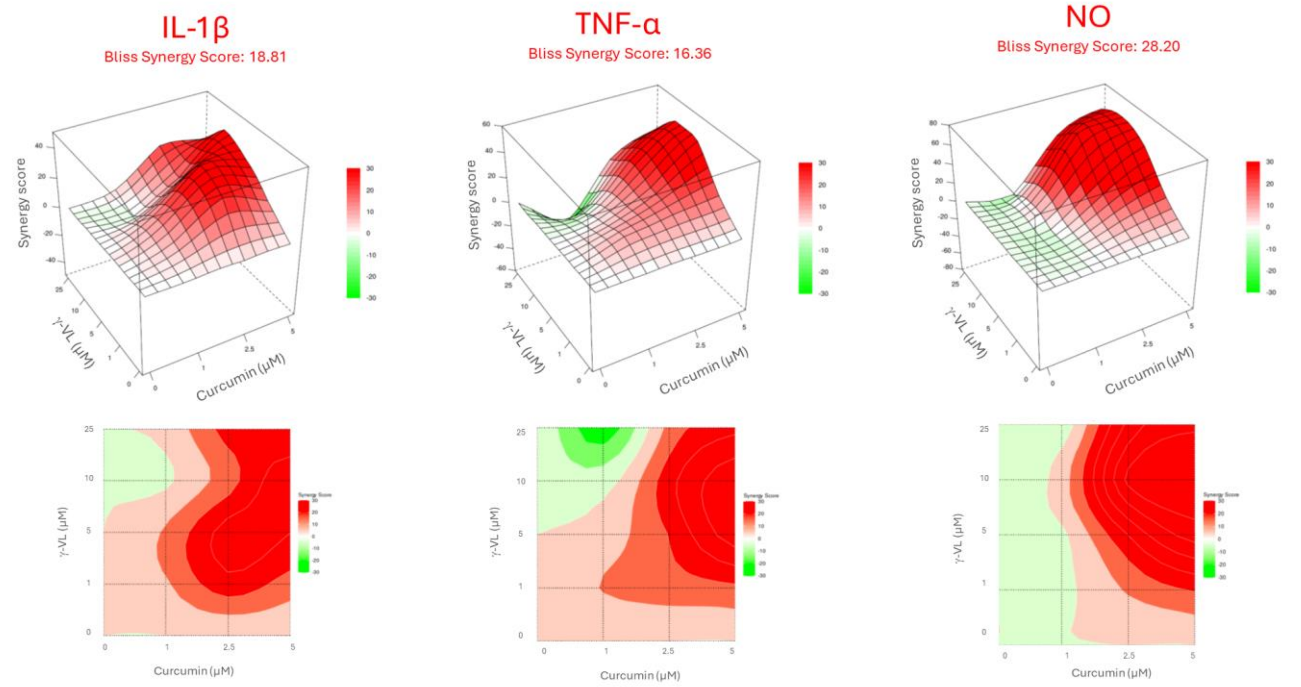

Figure S3. Concentration–response maps of pro-inflammatory mediator inhibition obtained with the tested curcumin and γ-VL combinations, according to Bliss model. The upper panel shows 3D surface, while the lower panel shows 2D contour. For each mediator, the average synergy score is indicated. Red color indicates synergism, while green color suggests antagonistic interaction.

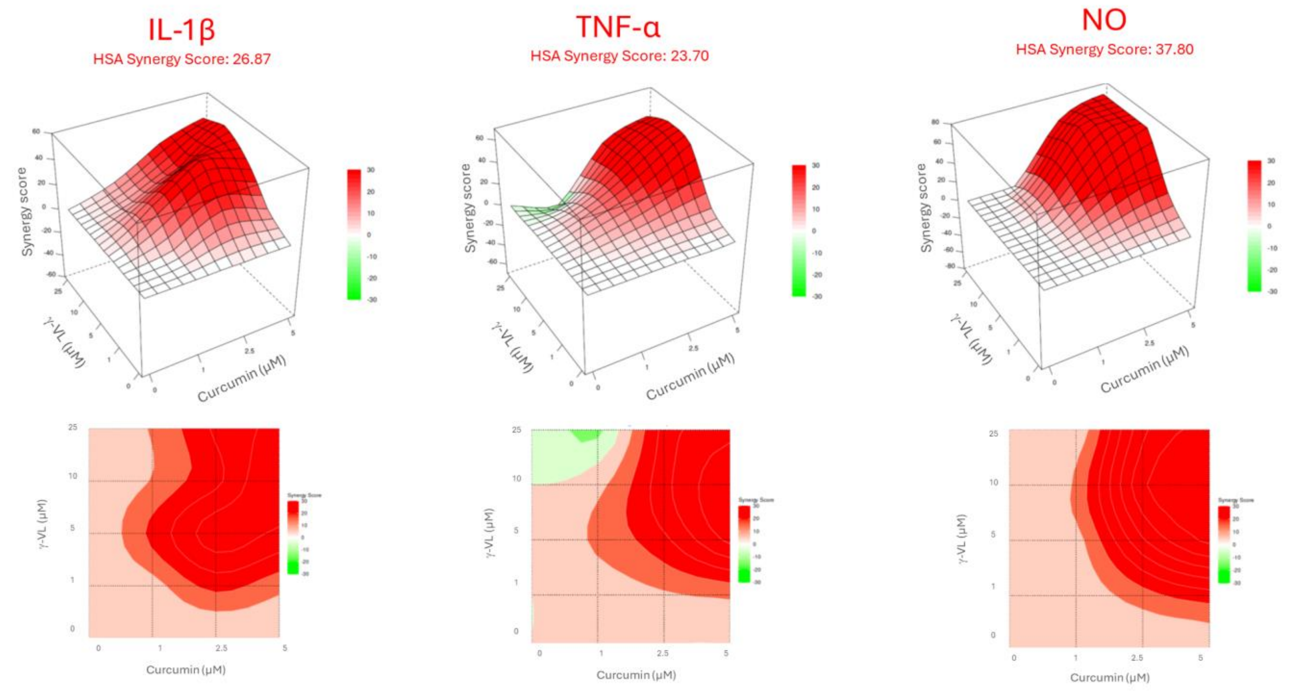

Figure S4. Concentration–response maps of pro-inflammatory mediator inhibition obtained with the tested curcumin and γ-VL combinations, according to HSA model. The

upper panel shows 3D surface, while the lower panel shows 2D contour. For each mediator, the average synergy score is indicated. Red color indicates synergism, while green color suggests antagonistic interaction.
